# Non-standard employment and mental health during the COVID-19 pandemic in Spain: a qualitative study: Eva Padrosa

**DOI:** 10.1093/eurpub/ckac131.268

**Published:** 2022-10-25

**Authors:** E Padrosa, M Julià, M Bolíbar, M Gutiérrez-Zamora, A Escrig-Piñol, The PWR Consortium

**Affiliations:** 1 ESIMar, Parc de Salut Mar, Universitat Pompeu Fabra, Barcelona, Spain; 2 SDHEd, IMIM, Barcelona, Spain; 3 GREDS, Universitat Pompeu Fabra, Barcelona, Spain; 4 Department of Sociology, Universitat de Barcelona, Barcelona, Spain; 5 JHU-UPF Public Policy Center, Universitat Pompeu Fabra, Barcelona, Spain

## Abstract

**Background:**

The COVID-19 pandemic entailed a deep economic crisis that affected working populations globally. However, non-standard workers (NSW, understood as workers with temporary contracts, not working full-time, self-employed or not paying taxes/making active pension contributions) were more severely hit than workers with more stable and protected jobs. The aim of this study thus was to explore the experiences of NSW during the pandemic and how these affected their mental health in Spain, one of the countries in Europe with the highest shares of temporary and self-employment and the one that applied the most restrictive containment measures during the first waves of the pandemic.

**Methods:**

As part of a larger multi-country study, 41 semi-structured interviews with NSW aged 25-55 were conducted between March-July 2021 and analyzed thematically.

**Results:**

Analyses revealed that job loss or insecurity and subsequent reductions in income were central to the experience of NSW during the pandemic in Spain, which affected their mental health negatively. Both the existing social protection framework and the policies deployed during the pandemic to outweigh these consequences were perceived by NSW as insufficient or could not access them due to their condition of NSW. This was particularly the case for self-employed and temporary agency workers. Moreover, NSW expressed that containment measures and reductions in income prevented them from engaging in activities to cope with such adversities, aggravating their mental health.

**Conclusions:**

These findings suggest that, in Spain, the pandemic intensified but also made more visible the defenselessness of NSW (especially self-employed and temporary agency workers) in terms of social protection in the event of sudden unemployment or reductions in income. This situation had harmful consequences for their mental health. Yet, new policies and measures fall short in fitting the necessities of an increasing share of the workforce.

**Key messages:**

• In Spain, non-standard workers are more vulnerable to and defenseless against sudden unemployment or reductions in income.

• The pandemic intensified this problem and put them at higher risk of suffering from poor mental health.

